# Accelerating research to practice by designing rigorous type 1 hybrid efficacy and effectiveness-implementation studies

**DOI:** 10.1017/cts.2025.10048

**Published:** 2025-06-03

**Authors:** Kathryn A. Hyzak, Alicia C. Bunger, Lisa Juckett, Daniel M. Walker, Bryan R. Garner

**Affiliations:** 1 Department of Physical Medicine & Rehabilitation, The Ohio State University, College of Medicine, Columbus, Ohio, USA; 2 The Center for the Advancement of Team Science, Analytics, and Systems Thinking in Health Services and Implementation Science Research, The Ohio State University College of Medicine, Columbus, USA; 3 Division of General Internal Medicine, The Ohio State University College of Medicine, Columbus, USA; 4 School of Health and Rehabilitation Sciences, The Ohio State University College of Medicine, Columbus, USA; 5 Department of Family and Community Medicine, The Ohio State University College of Medicine, Columbus, USA

**Keywords:** Implementation mechanisms, setting-intervention fit, implementation climate, hybrid effectiveness-implementation, research translation, implementation science

## Optimizing research-to-practice translation using implementation science

Implementation science has rapidly expanded over the past 25 years to improve the efficiency of research-to-practice translation. The field initially focused on conceptualizing implementation outcomes as distinct from clinical and service outcomes [1]. Early work centered on identifying implementation barriers and strategies to overcome them (e.g., training and facilitation). Today, there is growing emphasis on mechanisms of implementation strategies (i.e., how, why, and when they work) [2]. For innovations (i.e., pharmaceuticals, psychosocial interventions, and policies) to reach populations equitably and close the lengthy research-to-practice time lag, new approaches were needed to ensure that innovations are scaled while maintaining effectiveness. Hybrid studies, including type 1, type 2, and type 3 effectiveness-implementation studies [3], and more recently, DIeSEL (Dissemination, Implementation, effectiveness, Sustainment, Economics, and Level-of-Scaling) hybrid studies [4], have been codified to fill this need. However, limited guidance exists for generating insights about implementation in efficacy (i.e., whether the intervention works in ideal settings) or effectiveness (i.e., whether the intervention works in practice) stage hybrid trials. Optimizing hybrid studies as early as possible in the research translation continuum to produce strategic knowledge about the innovation and its implementation has potential to improve the efficiency of research translation, and ultimately, societal impact.

## Effectiveness-implementation hybrid studies

Carroll and Rounsaville (2003) first proposed that elements of intervention efficacy and effectiveness research be blended into a hybrid model to prepare for “real-world” application [5]. For example, they explained that teams might consider retaining randomization necessary for establishing intervention efficacy but diversify the delivery context or expand patient inclusion criteria to enhance generalizability. Curran et al. advanced this hybrid concept further by introducing effectiveness-implementation hybrid studies [6]. Indeed, with complexities of the health and social care system, and the frequent co-occurrence of biopsychosocial conditions throughout populations, scientists and healthcare professionals must rethink how we approach the research that ultimately impacts patient care.

Hybrid effectiveness-implementation studies include both an intervention effectiveness aim and an implementation aim in one study. A type 1 hybrid study is primarily focused on examining the effectiveness of an intervention on patient outcomes (e.g., symptoms, functioning) while also considering factors bearing on its implementation potential [3]. A type 2 hybrid usually has co-primary aims simultaneously examining the effectiveness of an intervention on patient outcomes and the effectiveness of the implementation strategy or strategies on implementation outcomes. A type 3 hybrid study is focused on examining the effectiveness of implementation strategies on implementation outcomes, while also gathering intervention effectiveness data.

Notably, hybrid studies have been recommended for use after the efficacy of the intervention has been established [3]. This recommendation stems from the requisite that the intervention has demonstrated safety and unintended consequences have been investigated. Nevertheless, the progression from efficacy to effectiveness is not necessarily linear or distinct, particularly for social, behavioral, and policy interventions [5,7]. Further, following the status quo research translation paradigm using linear progression from efficacy testing through pragmatic dissemination and implementation trials has resulted in the slow or no translation of interventions into practice settings, as well as reduced effectiveness as the intervention moves through the research-to-practice translation continuum [8]. With this inception of a much-needed scientific modernization, and long-overdue prioritization of health equity, social justice, and resource efficiency, we recommend consideration of dissemination, implementation, and sustainment by intervention trialists as early as possible in the research-translation continuum, including during efficacy testing. We contend that implementation can and should be investigated during the efficacy testing stage, and hence, these studies should be specifically designated as hybrid type 1 efficacy-implementation studies.

## Hybrid type 1 studies

Hybrid type 1 studies can be designed more rigorously to accelerate research-to-practice gaps and enhance pragmatic application. Opportunities exist to introduce implementation during intervention efficacy testing stages. For example, guided by the Reach, Effectiveness, Adoption, Implementation, and Maintenance framework (RE-AIM) [9], Bartholemew et al. used a type 1 hybrid study to investigate efficacy (rather than effectiveness) and implementation of the Comprehensive-TeleHarm Reduction intervention for increasing engagement in HIV prevention care [10].

Much more knowledge about implementation can be gained from more rigorously designed type 1 hybrids than what is currently being generated. Existing applications of type 1 hybrids generally focus on assessing determinants (i.e., What are the barriers and facilitators to implementation?) and perceptions about the intervention (i.e., Is the intervention acceptable?). These types of research questions likely stem from existing recommendations [3], published study examples, and/or insufficient availability of implementation research expertise in the workforce.

## Advancing type 1 hybrid studies

The growth of implementation research across diseases and conditions, contexts, and populations, as well as continual advancements to implementation concepts, necessitate boundary-pushing to keep pace with scientific developments and complexities of healthcare and patient needs. More recently, type 1 hybrid studies have started to investigate specific implementation determinants, like implementation climate, readiness for change, and innovation-values fit [11], which could be key mechanisms used to inform implementation strategy selection or refine implementation strategies to test in subsequent hybrid studies. Furthermore, although hybrid studies are distinct from pragmatic trials, key conceptual alignments can be leveraged for complementarity in efficacy stages. Specifically, type 1 hybrids include purposeful evaluation of implementation outcomes and contextual determinants, while pragmatic trials test interventions in the contexts where they are meant to be delivered, for example by leveraging existing personnel to deliver the intervention within the greater system and processes of care. Notably, the PRagmatic Explanatory Continuum Indicator Summary (PRECIS-2) Tool [12] can be used to design more pragmatic type 1 hybrid studies that leverage key delivery domains important for real-world application (e.g., broader patient inclusion criteria, flexibility in intervention delivery, existing organizational resources), while also examining implementation outcomes and specific theory-driven contextual determinants. In turn, this could lead to optimized type 2 hybrid studies that test implementation strategies and expedite the research-to-practice pipeline.

We assert that teams should work alongside healthcare providers, community partners, and patients to design interventions focused on equitability, which could reduce access and implementation barriers at later stages of research translation. Further, teams should design interventions to align with capacities, goals, needs, and values of the target setting and packaged for more effective dissemination to target audiences [11,13]. Moving beyond the design stage, engagement with partners should continue throughout the study to assess setting-intervention fit and contextual determinants that may impact intervention effectiveness and use, particularly given the dynamic nature of the implementation context. Furthermore, theories remain underutilized across intervention and implementation research. Implementation (e.g., Theory of Implementation Effectiveness) or classic theories (e.g., Theory of Planned Behavior) can guide more precise implementation aims and hypotheses that explain how and why phenomena occur [14]. By grounding implementation aims in theory, type 1 hybrid studies can advance the science of implementation through theory building, enhance generalizability of the phenomena to other contexts, and inform preliminary causal models of implementation process mechanisms [2,14,15].

Further, intervention trialists often use implementation strategies during efficacy and effectiveness trials (i.e., training, audits, feedback) without acknowledging or reporting these strategies, which prohibits replicability [16] or delays testing specific strategies to improve implementation, sustainment, and scale-up. In Table 1, we provide new, specific recommendations for maximizing type 1 hybrid studies based on the implementation science literature.

**Table 1.** Recommendations for hybrid type 1 efficacy and effectiveness-implementation studies

Current Problem	Recommendation	Rationale	Example(s)
1. Limited consideration of setting-intervention fit when developing and/or selecting an intervention for testing as part of a hybrid type 1 study.	Design interventions in collaboration with community partners, and evaluate setting-intervention-fit within routine practice settings over time to account for the dynamic nature of the implementation context. Design interventions that align with the capacities, goals, needs, and values of the intended population and implementation setting.	Interventions are a good fit to the extent that they are considered equitable, fundable, implementable, retainable, sustainable, scalable, and timely for the practice setting.	Garner et al. (2022) longitudinally assessed the setting-intervention fit (e.g., the extent to which the intervention is fundable, implementable, retainable, sustainable, scalable, and timely) of the Positive Health Check intervention across four HIV treatment clinics [11].
2. Limited specification of the intervention’s core functions.	Develop a preliminary fidelity measure based on the identified functions of the intervention, and pilot test a fidelity monitoring approach in the clinical trial. Explicitly state the core functions of clinical interventions as well as the methods for documenting how the intervention is tailored to the local context.	Fidelity measures are highly specific to an innovation and can take time to develop and refine. Beginning the development of a fidelity measure and approach to monitoring fidelity during efficacy and effectiveness studies is needed to monitor fidelity during the trial, establish evidence that the intervention can be delivered as intended, and offer opportunities to pilot test data sources that will be needed for future implementation studies. Specifying the core functions of a clinical intervention enables teams to monitor fidelity, and tracking variations in how the intervention is tailored can help teams determine which intervention adaptations are associated with better outcomes	Although not a hybrid type 1 study, Kirk et al. (2021) describe their systematic process of identifying the core functions and forms of an intervention designed to increase physicians’ referrals to hospice for nursing facility residents [17]. This process was characterized by six steps (e.g., analyzing existing materials, interviewing staff, mapping forms onto their associated core functions) that led to the identification of two core functions and their prevailing forms (i.e., adaptations).
3. Limited utilization of implementation science theory.	Utilize implementation science theory or theories to develop the implementation aim of a hybrid type 1 study.	Theories can help guide a more precise implementation aim and hypothesis(es), which can be used to specify potential mechanisms of change.	Guided by the Theory of Implementation Effectiveness [15], which posits implementation climate (the extent to which implementation is expected, supported, and rewarded) as a key mechanism of change, Garner et al. (2022) examined changes over time in implementation climate using a longitudinal mixed-methods approach [11].
4. Limited implementation strategy specification.	Specify each discrete, multifaceted, or blended strategy in accordance with the recommendations provided by Proctor et al. (2013) [16].	Specification of each discrete, multifaceted, or blended strategy used as part of a hybrid type 1 study is important for transparency and replication, as well as refining strategies to test in a hybrid type 2 study.	Bartholemew et al. (2024) will use a trial logbook and clinic observation data to track strategies used during their hybrid type 1 trial [10].
5. Limited consideration of implementation and/or intervention costs.	Examine the economic costs of the intervention and its comparison, as well as the costs associated with implementation.	Costs of an intervention and the resources to implement the intervention can affect feasibility, perceptions of acceptability, implementation, and sustainability.	In their hybrid type 1 trial Bartholomew et al. (2024) will evaluate the cost-effectiveness of two comparative intervention models and their implementation using macro- and micro-costing techniques [10].
